# Exergaming Using Postural Feedback From Wearable Sensors and Exercise Therapy to Improve Postural Balance in People With Nonspecific Low Back Pain: Protocol for a Factorial Pilot Randomized Controlled Trial

**DOI:** 10.2196/26982

**Published:** 2021-08-26

**Authors:** Anita Meinke, Rick Peters, Ruud Knols, Walter Karlen, Jaap Swanenburg

**Affiliations:** 1 Mobile Health Systems Lab Institute of Robotics and Intelligent Systems, Department of Health Sciences and Technology ETH Zurich Zurich Switzerland; 2 Nursing and Allied Health Profession Office Physiotherapy Occupational Therapy University Hospital Zurich Zurich Switzerland; 3 Directorate of Research and Education Physiotherapy Occupational Therapy Research Center University Hospital Zurich Zurich Switzerland; 4 Institute of Human Movement Sciences and Sport Department of Health Sciences and Technology ETH Zurich Zurich Switzerland; 5 Faculty of Medicine University of Zurich Zurich Switzerland

**Keywords:** low back pain, exercise therapy, postural balance, postural feedback, motor control, fear of movement, exergame, randomized controlled trial, physical activity, smartphone, sensors, activity tracker, mobile phone

## Abstract

**Background:**

Physical exercise is a common treatment for people with low back pain (LBP). Wearable sensors that provide feedback on body movements and posture during exercise may enhance postural balance and motor control in people with LBP.

**Objective:**

This study aims to investigate whether physical exercising with postural feedback (EPF) improves postural balance, motor control, and patient-reported outcomes in people with LBP.

**Methods:**

The study was an assessor-blinded 2×2 factorial trial. We planned to recruit 80 participants with nonspecific LBP who did not receive treatment for LBP. In addition, we aimed to recruit 40 patients with chronic, nonspecific LBP who were receiving exercise therapy (ET) at the University Hospital Zurich. Both ET patients and participants without treatment were randomized to receive either an additional EPF intervention or no additional intervention. This resulted in four different combinations of interventions: ET+EPF, ET, EPF, and no intervention. The participants underwent outcome assessments at inclusion (T1); 3 weeks later, at randomization (T2); after an intervention period of 3 weeks with a predefined exercise schedule for participants receiving EPF (T3); and after an additional 6 weeks, during which participants assigned to the EPF groups could exercise as much as they wished (T4). Patients receiving ET completed their regularly prescribed therapies during the study period. Balance was assessed during quiet standing on a force platform, and motor control was assessed during a lifting task and a waiter’s bow task. Physical activity was recorded using an activity tracker and the participants’ mobile phones during the study. The predefined EPF schedule consisted of nine sessions of 20 minutes of exercise with a tablet and inertial measurement unit sensors at home. Participants performed a series of trunk and hip movements and received feedback on their movements in a gamified environment displayed on the tablet.

**Results:**

The first participant was recruited in May 2019. Data collection was completed in October 2020, with 3 patients and 32 eligible people without therapy who passed the eligibility check.

**Conclusions:**

Although it will not be possible to investigate differences in patients and people without other therapies, we expect this pilot study to provide insights into the potential of EPF to improve balance in people with LBP and adherence to such interventions.

**International Registered Report Identifier (IRRID):**

DERR1-10.2196/26982

## Introduction

### Background

Digitalization is transforming health care and has the potential to increase the effectiveness of interventions. Novel tools can complement traditional therapies, offer new training formats, and allow different settings for providing interventions. As the number of those affected by low back pain (LBP) exceeds 500 million people worldwide [1] and self-management is recommended [2], digital tools may have a beneficial impact in reducing the total burden. It has been suggested that changes in spinal motor control can contribute to LBP persistence [3-5]. Digital tools that quantify trunk movements and provide feedback during physical exercises may be efficient in supporting people with LBP to improve their motor control of the trunk. As studies have demonstrated that fatigue of the trunk muscles [6] and trunk stiffness [7-9] affect postural balance, we hypothesize that exercising with postural feedback (EPF) on trunk movements can improve postural balance in people with LBP. Exergaming has shown promising effects in initial applications for musculoskeletal conditions [10]. However, available studies cover different conditions and interventions, and because of this heterogeneity, the results could not be numerically integrated in a recent review [10]. It was concluded that this field should be further explored [10]. In a review, it was shown that exercise interventions supported by digital tools for people with chronic LBP were successful only when provided in conjunction with other treatments and tested against the unaccompanied other treatment but not when compared by itself against another condition [11]. Similarly, improvements in postural balance may depend on being combined with parallel rehabilitative interventions. Therefore, this study included patients who received regular prescribed therapies, including exercise therapy (ET), and a group of participants who did not receive therapy for LBP.

### Objectives

The primary research objective is to investigate whether a home exercise program with feedback on trunk movements can improve postural balance and other health-related outcomes in people with LBP. Secondary research objectives refer to the adherence to EPF, subgrouping participants with LBP based on digitally acquired data, comparison of objectively monitored activity data with self-reported activity data, and the relationship between fear of movement and postural balance. To keep the protocol concise, only the primary research objective is described in detail in this paper, and additional research questions will be addressed in future publications.

The hypotheses corresponding to the first research objective and the primary outcome of postural balance are as follows: EPF improves *postural balance*, and the improvements in *postural balance* because of EPF among participants not receiving therapy are more beneficial than the improvements in postural balance in ET patients receiving ET. Secondary outcomes include indicators for *motor control*, *pain intensity*, *disability*, *quality of life*, and *fear of movement*.

The intervention group is compared with a control group that did not receive an additional intervention. Potential difficulties in recording and comparing adherence in equal quality in groups with and without a digital tool played a role in this decision.

### Postural Balance in LBP

Postural balance is different in people with LBP in comparison with the postural balance of healthy participants [12-14], although balance may not be changed in the same manner across all people with LBP [14]. In studies where passive trunk stiffness was experimentally manipulated using corsets or lumbar belts, balance was reduced in different tasks, for example, when regaining balance after being released from an unstable, forward-leaning position [7] or during seated balancing on a labile surface [8]. However, in another study using a similar seated balancing task, only voluntary activation of trunk muscles led to faster sway, whereas stiffness caused by a lumbar belt did not lead to a reduction in balance [9]. In addition, a systematic review reported that trunk muscle fatigue resulted in faster sway during standing [6]. This indicates that deviating motor control of the trunk may play a significant role in the altered postural sway characteristics observed in people with LBP. Further evidence provided by a meta-analysis indicates that postural balance can be enhanced in older adults through balance exercises, although no changes were found for interventions targeting strength or comprising different training approaches [15].

For people with LBP, the effects of exercise on postural balance have been documented in several studies [16-21]. An effect on postural balance was found in some of these studies comparing different groups [18-20] but not in other studies [21]. The results of the study by Lomond et al [17] indicated that different interventions can even result in diverging changes in the same outcome. Although the included populations, tasks, postural balance parameters, and interventions vary considerably in the abovementioned studies, they show that postural balance can potentially be modified by exercising interventions in people with LBP.

### EPF for People with LBP

Some feedback is naturally available during movement, for example, as visual or proprioceptive input [22]. Additional information that needs to be obtained from external sources can be measured or reported by another person and may be used for the acquisition and correction of movement patterns [22]. For people with LBP, additional feedback on trunk movements might be effective in improving motor control of the trunk and, thus, postural balance.

Two systematic reviews reported that different types of digital feedback, for instance, from electromyography [11,23] or ultrasound data [23], on muscle activity have been investigated more frequently for their use in exercising interventions for LBP than postural feedback from digital tools. Some studies have already investigated exercise interventions on postural feedback on trunk movements in clinical settings in people with LBP [24-27]. In one study, it was concluded that the intervention increased range of motion and movement speed [25], although it is unclear from the reporting whether these results refer to a comparison between groups or a change over time. Kent et al [24] found no group differences in range of motion, and another study investigating effects on movement control impairment did not find a significant difference between groups [27]. As the number of studies seems to be limited, more research could help to better estimate the potential of such interventions on movement characteristics and postural balance in people with LBP.

## Methods

### Overview

The SPIRIT (Standard Protocol Items: Recommendations for Interventional Trials) checklist [28-30] for this protocol can be found in Multimedia Appendix 1.

### Study Design

The study was conducted at the Physiotherapy Occupational Therapy Research Department at the University Hospital Zurich (UHZ). The Cantonal Ethics Committee Zurich (Business Administration System for Ethics Committees 2018-02132) approved this study.

**Figure 1:**
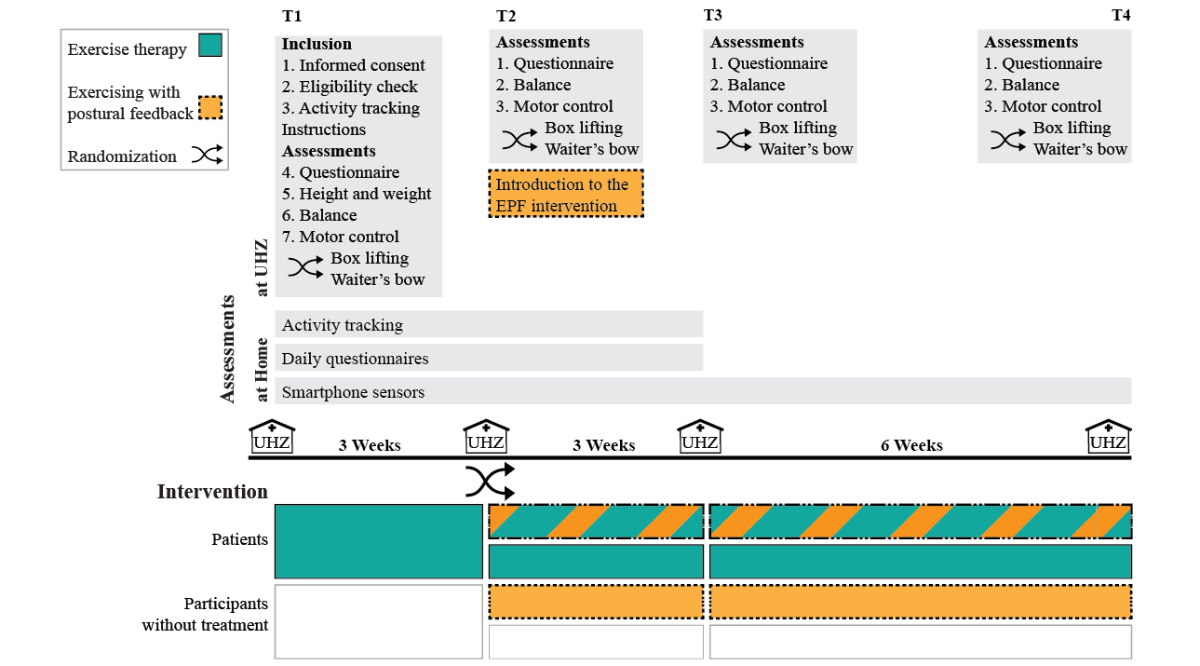
Study procedures. Procedures displayed in orange are specific for participants receiving exercising with postural feedback. Procedures in green are specific to exercise therapy patients receiving exercise therapy. EPF: exercising with postural feedback. UHZ: University Hospital Zurich.

The study is a 2×2 factorial randomized controlled superiority trial, and it has been summarized in Figure 1. We aimed to recruit 40 patients with chronic, nonspecific LBP receiving ET training at the UHZ and 80 additional participants currently not receiving any treatment for LBP. Patients in ET continued with their regular prescribed therapies during the study participation, whereas participants without therapy for LBP were requested not to seek treatment for LBP while participating. At the second appointment (T2) at 3 weeks from inclusion, the patients and the participants without treatment for LBP were randomized to either receive or not receive the EPF intervention. This setup resulted in 4 groups: ET+EPF, ET, EPF, and no intervention. Study participants who did not receive EPF during the study (the ET alone and no intervention groups) had the possibility for EPF for 3 weeks after the completion of their last assessment (T4). Assessments of primary and secondary outcomes at the UHZ took place at inclusion (T1), 3 weeks after randomization (T2), after completion of the 3-week intervention period (T3), and after a second intervention period of 6 weeks without a predefined exercise schedule (T4).

### Randomization

Participants were randomized to the EPF intervention or control condition while completing the second assessment at T2 with the outcome assessor. Stratified block randomization (1:1 assignment ratio) was used, with stratification by height and block sizes of 2 and 4. AM generated the randomization lists in R using the blockrand package (Snow, 2013, version 1.3) and randomized the participants using the randomization tool in Research Electronic Data Capture (REDCap) [31] hosted at Eidgenössische Technische Hochschule Zurich (ETH Zurich). Outcome assessors were blinded to the randomization results but other study staff members were not. The conditions under which group assignments could be revealed to outcome assessors were not specified.

### Eligibility and Consent

The participants received a detailed information document that was sent via email or was printed in advance, discussed the study participation, and provided informed consent to AM. On the consent form, the participants could separately indicate whether they wished to contribute with their data to answer other research questions that were not defined. Eligibility was determined according to the criteria listed in Textbox 1 after the participants provided informed consent. If interviewing the study participants with respect to the criteria was insufficient to determine eligibility, the research scientist (AM, psychologist by training) consulted a physiotherapist team member (RP, RK, or JS). As ET is usually but not always provided in conjunction with physiotherapeutic treatments, additional treatments in combination with ET were not restricted. Participants without therapy were not allowed to undergo other treatments for LBP during the study. Leisure-time sports activities were not restricted.

**Eligibility criteria for patients receiving exercise therapy and participants without treatment for low back pain.**

**Inclusion Criteria**

- Patients receiving exercise therapy
  Patients with chronic nonspecific low back pain
  Patients undergoing medical training therapy (exercise therapy) at the University Hospital Zurich
  Adult male and female participants (aged ≥18 years)
  Informed consent as documented by signature
- Participants without treatment
  Nonspecific low back pain
Receiving no therapy or medical treatment for the last 6 months
  Adult male and female participants (aged ≥18 years)
  Informed consent as documented by signature

**Exclusion Criteria**

- Patients with specific causes for low back pain
- Radicular syndrome
- Unable to participate currently in the program due to pain
- Pregnancy
- Medication reducing postural balance
- Uncorrected heavy visual impairment
- Allergy to adhesive tape
- Unable to understand and communicate in German or English

### ET Intervention

At UHZ, patients begin with ET after completing nine physiotherapy appointments. ET includes two sessions of 60 minutes per week for a period of 12 weeks. The therapy starts with an introductory session in which the physiotherapist develops an individually tailored exercise plan based on the personal needs of the patient, and the therapy ends with a debriefing session. Patients are contacted via phone when they miss an ET appointment. During the therapy sessions, patients follow their exercise plans independently but under the supervision of an experienced physiotherapist in a group setting at the UHZ. The primary goal of ET is to increase the general load-bearing capacity of the lumbar-pelvic region. ET is focused on functional exercises such as front raises or squats and includes exercising with standard fitness equipment such as ergometers and fixed and free weights at a progressive resistance.

### EPF Intervention

This section summarizes the information required by the Template for Intervention Description and Replication (TIDieR) checklist [32]. Other information regarding the intervention rationale and assessment of adherence can be found in the corresponding sections. Participants who were randomized to the EPF intervention received the Valedo home system (Hocoma AG) and a tablet (MediaPad T5, Huawei) for exercising at home. The participants were asked to complete nine exercise sessions at regular intervals within the 3-week period between T2 and T3. The sessions consisted of 10 predefined exercises of 2 minutes each, resulting in a total exercise time of 180 minutes. Starting from T3 to T4, participants in the EPF groups could continue to exercise without adhering to a defined schedule and choose freely among all available exercises.

**Figure 2:**
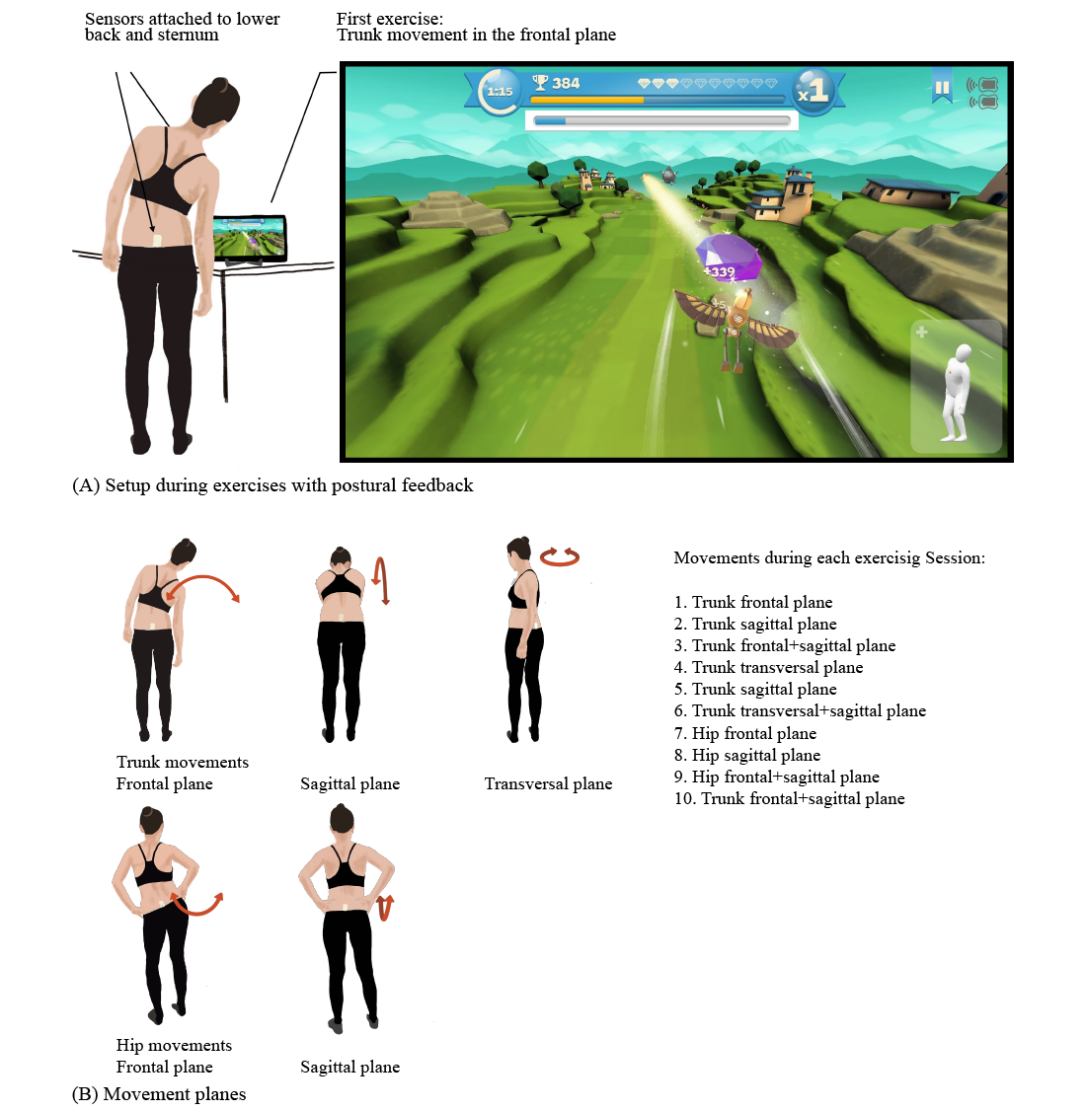
EPF Exercises given to participants to complete at home. (A) Setup of the home exercising intervention and screen view of the Valedo app (with permission from Hocoma AG). (B) Movements practiced during the exercising with postural feedback intervention.

After the assessments at T2, AM introduced the EPF intervention to the participants randomized to the EPF groups. This included completing the first exemplary exercise and familiarization with the handling of the tablet and the Valedo. The participants created a user profile and were guided through an assessment of the range of motion of the trunk and hip. This information is used by the software to adapt the extent of movement required for each user [33]. Furthermore, the participants were instructed on how to exercise at home and received a document summarizing the instructions, including the list of exercises to complete (Figure 2) and the user manual of the device [33].

The Valedo system consists of two inertial measurement unit sensors and an accompanying app that provides different exercises within a gamified environment. For exercising, the IMUs are attached using either medical adhesive tape or belts to the lower back and the sternum (Figure 2) and they measure trunk and hip movements on different planes. The posture and position of an avatar on the screen continuously show the player’s movements relative to the movement goal. This goal is either displayed as a white track the participant tries to follow or as crystals that are collected by performing the required movements as accurately as possible. In addition to this visual feedback, the player receives auditory feedback when matching the track precisely and gathering crystals. After completion of an exercise, the last exercise is shown in a ranking of the 10 best previous attempts of the player. For this study, the first nine exercises within the Valedo exercising environment were selected, whereas the third exercise was repeated once. Each exercise required a trunk or hip movement on a single plane or alternating movements on different planes. It was not specified in advance how the intervention would be adapted if this would become necessary. Adherence was analyzed after the completion of the study. The participants were instructed to contact the study coordinator in case of difficulties.

### Outcomes

#### Postural Balance

**Figure 3:**
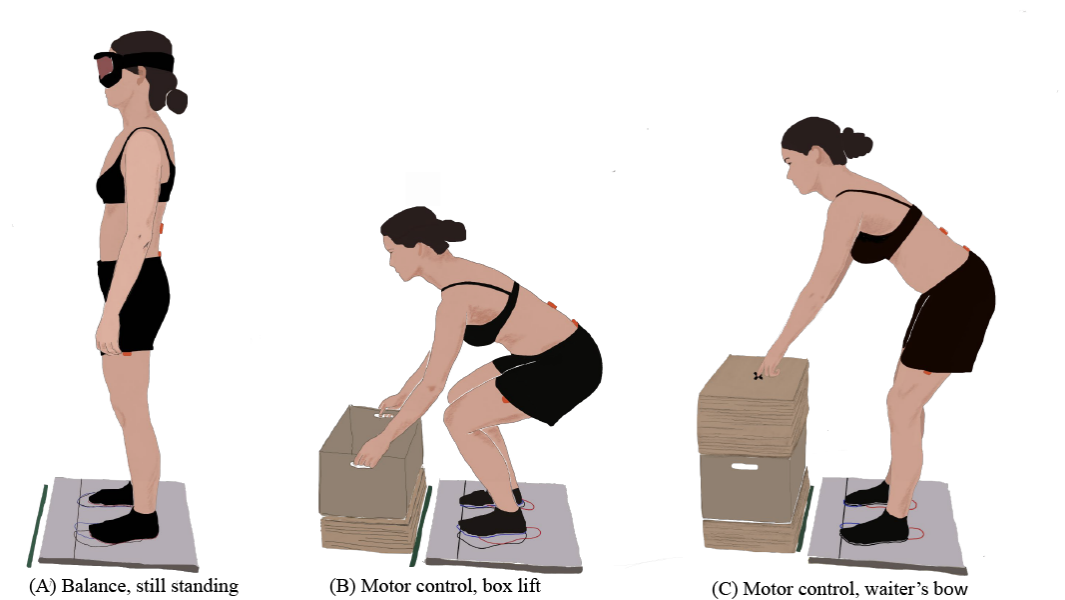
Movement tasks. (A) Balance: standing still, (B) motor control: box lift, and (C) motor control: waiter’s bow.

Postural balance was assessed through center of pressure (COP) recordings during bipedal stance (Figure 3). The design of the balance assessment was based on the recommendations of Ruhe et al [34]. COP data were collected at a rate of 100 Hz using an Accusway Plus force platform (Advanced Medical Technology Inc). Each recording took 120 seconds and was repeated four times at each assessment visit. Study participants were instructed to stand on the force platform “as still as possible” [34] with their arms hanging at the side. During the recording, the participants kept their eyes closed and wore an opaque mask. To standardize the foot position across all assessment repetitions and assessment visits, the participants were asked to step on the force platform and stand in a comfortable foot position before the first COP recording. The foot position was then marked on a foil and kept for all following postural balance recordings of the participant. Differences in COP sway in the anterior-posterior direction between T3 and T2 will be analyzed as the primary outcome. In addition, other sway characteristics, such as sway in the mediolateral direction and COP velocity, will be examined.

#### Motor Control

Assessments of lumbar spine and hip angles during a box lifting and waiter’s bow task were used as indicators of motor control. The tasks, setup, and instructions of the motor control assessments were adapted from the study by Matheve et al [35], where the assessments demonstrated good reliability. Unlike Matheve et al [35], we did not adjust the lumbar spine posture to fit an ideal posture before the assessments because we assumed that this procedure could increase the task difficulty selectively for participants with a stronger deviation. Furthermore, we added an assessment of balance during the tasks, only allowed the participants to practice each task once before the assessment, and asked participants to report their pain during the practice run.

Lumbar spine and hip angles were derived from measurements using the Valedo Pro assessment tool (Hocoma AG). Three IMUs were attached to the skin with medical adhesive tape at the S1 and L1 spinous processes and at the left thigh, 20 cm on top of the lateral femoral condyle, analogous to the study by Matheve et al [35]. For positioning the IMUs correctly, the outcome assessors palpated the back and knee of the participant. The orientation of the sensors was sampled at a rate of 50 Hz, and the data were transmitted via Bluetooth to a laptop. During the motor control assessments, additional data on postural balance were collected using the force platform. The outcome assessor randomized the order of the motor control tasks before each assessment visit by flipping a coin. The participants repeated each motor control task five times. The outcome assessor demonstrated correct and false task performance before the participants practiced each task once at each assessment visit. The participants then reported their pain during the practice movement as a safety outcome. Before the performance on each task was recorded for the first time, the outcome assessor instructed the participants to stand on the force platform with their feet parallel, with their toes on a marked line, and at a width that the participants perceived as comfortable for the task. The outcome assessor recorded the foot position on the foil to standardize the position across all task repetitions and assessment visits for each participant.

To perform the box lifting task (Figure 3), the participants started in an upright standing position, with the feet parallel and the toes touching the marked line on the force platform. The participants lifted the box, held it in a standing position, and put the box back down. The participants were instructed to maintain the same neutral position of the lumbar spine during the task. The box was 40 cm wide, 30 cm deep, 24 cm high, and 3.5 kg heavy, and the position was individually adjusted for each participant (upper front edge at a level of 10 cm below the apex of the kneecaps and 15 cm in front of the toes).

For the waiter’s bow task (Figure 3), the participants stood with their feet parallel and toes on the line on the force platform. The outcome assessor asked the participants to bend forward using their hips and touch a marking in front with the tip of the index fingers and return to the starting position. The participants were instructed to keep their lower back in a neutral alignment and not to bend the knees during the task. The placement of the marking was adapted to each participant (10 cm above the base of the kneecap and 30 cm in front of the toes). Outcome assessors were trained on the assessment protocol and tools before performing any assessments.

#### Questionnaires

The participants completed a series of questionnaires at each assessment visit (Table 1) before the balance and motor control assessments. The questionnaires were administered on a laptop in English or German via REDCap surveys [31]. Pain intensity, physical functioning, and health-related quality of life were assessed as secondary outcomes, as these constructs have been proposed for general use in clinical trials on nonspecific LBP to enhance comparability among different studies [36]. Pain intensity was reported on an 11-point numeric rating scale (NRS; 0=no pain and 10=worst imaginable pain) referring to average pain during the previous week [37]. In a recent review, the results regarding the measurement characteristics of the NRS were found to be mostly mixed, and the measurement error was rated as too high [38]. Nevertheless, the NRS was selected based on the recommendation for widespread inclusion of the NRS in clinical trials on LBP [37].

**Table 1:** Overview of the questionnaires used in the study.

Outcome	At assessment visits					Daily	
	T1^a^	T2^b^	T3^c^	T4^d^	Morning		Evening
Demographic data	✓						
History of LBP^e^	✓						
Pain intensity: NRS^f^	✓	✓	✓	✓			
Disability: RMDQ^g^	✓	✓	✓	✓			
Quality of life: WHOQOL-BREF^h^	✓	✓	✓	✓			
Fear of movement: TSK-11^i^	✓	✓	✓	✓			
Movement-specific fear ratings	✓	✓	✓	✓			
Depression: PHQ-9^j^	✓	✓	✓				
Self-efficacy chronic disease: SES6^k^	✓	✓	✓	✓			
General self-efficacy: GSES^l^	✓						
Personality dimensions: BFI-10^m^	✓						
Amount of sleep					✓		
Sleepiness: KSS^n^					✓		
Physical activity: adapted from IPAQ^o^							✓
Pain intensity							✓

^a^T1: Assessment Time 1.

^b^T2: Assessment Time 2.

^c^T3: Assessment Time 3.

^d^T4: Assessment Time 4.

^e^LBP: low back pain.

^f^NRS: numeric rating scale.

^g^RMDQ: Roland Morris Disability Questionnaire.

^h^WHOQOL-BREF: World Health Organization Quality of Life-short version.

^i^TSK-11: Tampa Scale of Kinesiophobia-11.

^j^PHQ-9: Patient Health Questionnaire-9.

^k^SES6: Self-Efficacy for Managing Chronic Disease 6-Item Scale.

^l^GSES: General Self-Efficacy Scale.

^m^BFI-10: Big Five Inventory-10.

^n^KSS: Karolinska Sleepiness Scale.

^o^IPAQ: International Physical Activity Questionnaire.

Physical functioning was assessed using the Roland Morris Disability Questionnaire, which consists of 24 items [39,40]. Participants check all items that apply to their situation on the day of assessment [39,40]. The Roland Morris Disability Questionnaire is comparable with other established questionnaires [41], is reliable [39,40] and valid [39], and is suggested as one of the standard questionnaires to be used in clinical trials investigating LBP [37]. Quality of life was assessed using the short version of the World Health Organization Quality of Life Questionnaire, which consists of four subscales formed by 26 items [42]. A large study including data from different language versions and nations indicates that the World Health Organization Quality of Life Questionnaire-short version is a reliable tool for assessing quality of life [43]. Fear of movement was assessed using an 11-item version of the Tampa Scale of Kinesiophobia [44,45]. The adequacy of the psychometric characteristics of the Tampa Scale of Kinesiophobia with 11 items was confirmed for the English version [44,46] and a German translation [45]. These results were obtained from participants with LBP [44,45] and from a study that included participants with diverse pain conditions [46]. In addition, participants were asked to rate six movements (flexion, extension, sideways bending, rotation, lifting, and stretching) on slider scales (0=totally disagree and 100=totally agree) with respect to the harmfulness of the movement, pain, and how carefully participants would execute each movement. The movements were visualized using small icons. These questions were inspired by the format of the photograph series of daily activities-short electronic version [47] and the suggestion by Pincus et al [48] to consider painfulness and avoidance in addition to fear.

Instruments not listed as outcomes under the primary research question were the Patient Health Questionnaire-9 for assessing depression [49,50], Self-Efficacy for Managing Chronic Disease 6-Item Scale [51,52], the General Self-Efficacy Scale [53-55], and the Big Five Inventory-10 [56].

#### Activity Assessments

During the first 6 weeks of the study, the participants wore an activity tracker [57] and completed a short questionnaire on their mobile phone every morning and evening. The activity tracker was worn on the wrist of the nondominant arm or on the ankle in cases where the profession of the participant did not allow this. The tracker recorded activity counts at a sampling rate of 1 Hz. The participants were instructed to wear the tracker continuously, night and day, except when taking a shower or swimming. The questionnaire in the morning contained questions on estimates of bedtime, wakeup time, actual sleep duration, and quality of sleep. Sleepiness was assessed using the Karolinska Sleepiness Scale [58]. The questions in the evening were adapted from the International Physical Activity Questionnaire [59,60] and referred to the time spent in walking, sitting, and high and moderate amount of physical activity on that day. Furthermore, the participants were asked to rate the pain intensity during the day on a slider scale.

For the entire study duration, sensor data of the phone were collected from participants who agreed to the collection of these data and had a mobile phone with an Android operating system. The data collection was dependent on the availability of sensors in the phone and included acceleration and speed readings of the GPS location. In addition, data on the use of the app that provided the questionnaires were recorded.

#### Baseline Data and Adherence

General demographic data such as age, gender, education, occupation, and data on the participants’ history of back pain were collected using the questionnaire at T1. Participants' height and weight were measured using a personal scale and a bar before the first balance assessment. Completed exercises including timestamps were exported from the Valedo app and will be used to quantify adherence to the intervention. For patients in ET, data routinely assessed by physiotherapists at initiation of treatment, such as information on the diagnosis and dates of ET and physiotherapy appointments, were recorded.

#### Data Management

Electronic case report forms and questionnaire data and exercise adherence data were stored in REDCap [31]. Postural balance, motor control, and activity tracker data were stored on a protected network drive at ETH Zurich. Data from the activity tracking app were stored on a secured server (geographic location: CH, EU, or DE). Only staff members involved in the study had access to the study data and had to keep the data confidential. Data were saved with ID numbers and not with participant names. Electronic case report forms were double-checked for completeness by the principal investigator (JS). No data monitoring committee was established, but an external monitor reviewed the conduction and completeness of the study data. Parts of the data were reviewed while the trial was in progress, but intervention effects were not analyzed before the end of the study was determined. After trial completion, all data will be available to study team members from the UHZ and ETH Zurich. The results of the trial will be presented in a peer-reviewed journal.

#### Harms

The study was deemed low risk, as the Valedo home is a certified medical device and exercising is well established as a treatment for LBP. At the assessment visits, the participants were asked how they felt, and adverse events were documented on REDCap [31].

### Recruitment

ET patients were recruited at UHZ. Potentially eligible patients were contacted in person by the study personnel or physiotherapists, or they responded to flyers. The study personnel approached the patients at the initial appointments to keep the amount of therapy the patients had already received when entering the study similar. Participants without therapy for LBP were recruited by advertising on digital channels, word of mouth, and flyers. Participants received initial information on the study before the first assessment visit on the phone, either via email or in person. Reasons for dropping out were documented when specified by participants, and dropouts were not replaced. Email reminders were sent before assessment visits.

### Sample Size

Power was estimated using Gpower [61] using the *Linear multiple regression: Fixed model, single regression coefficient* menu. As this is a pilot study and we did not have data available to estimate the effect, we planned with an effect size of f^2^=0.063 and three predictors in the model to account for both factors and the interaction effect. The primary hypotheses refer to the effect of the EPF assignment on postural balance and the interaction effect of EPF with treatment status (patients in ET or no treatment); therefore, α was set to .025. To detect this effect at a power of 0.6, it would be necessary to include 80 participants. The number of patients receiving ET training at the UHZ is limited, whereas recruitment of more participants with LBP but not in therapy seemed feasible. Owing to these limitations, we planned to include 40 patients and 80 participants without therapy to account for dropouts.

### Statistical Methods

We will test the primary hypotheses using multiple regression models (with the two factors patient or participant and EPF or no EPF and their interaction term as predictors, and T3−T2 differences as outcome) or nonparametric equivalent tests where required. We will use intention-to-treat analysis to examine intervention effects and replace missing values at T3 if less than 10% (12/120) of the data are missing. The method of replacing missing data has not been defined in advance. We will explore other assessment visits between T1 and T4 using multilevel models. Analyses related to other questions not described in this manuscript are not listed.

### Amendment

This paper refers to protocol version 3, August 31, 2019. Few major changes have been made since the beginning of the study. To facilitate recruitment, the period participants had to be without treatment before inclusion was reduced from 1 year to 6 months and participants randomized to the control condition received the EPF program after study completion. In addition, the research questions referring to fear of movement and movement-specific fear questions were added. The described changes were approved by the Cantonal Ethics Committee Zurich in September 2019 and were implemented accordingly.

## Results

The first participant was recruited in May 2019. Owing to the COVID-19 pandemic, the trial was paused from March to May 2020. In October 2020, data collection was concluded, with 38 participants providing informed consent. Of those participants, 3 patients and 32 participants passed the formal eligibility check and completed T1. The trial ended with the time that had been allocated for the study conduction, not based on interim analyses. Data analysis is ongoing.

## Discussion

### Principal Findings

This study will provide information on how a digital intervention with feedback from IMUs on trunk movements affects postural balance and other outcomes in people with LBP. Daily questionnaires and continuous activity tracking will enable us to explore the development of pain intensity and other physical activities of each participant during the study in detail. The second intervention period without a predefined exercise schedule will give hints to the transferability of the results to a less controlled setting.

Within the allotted time, fewer participants could be recruited than initially planned, and only a few patients in ET could be recruited. Therefore, it will not be possible to compare the effects of the intervention between patients in ET and people with LBP who are not in treatment. Furthermore, when interpreting the results, it should be considered that no sham intervention was provided to the participants without therapy. Thus, general treatment-related factors are different among these groups.

The use of movement feedback from wearable sensors in exercise interventions is a promising approach to improve interventions. The detailed properties of the feedback given are likely to have an impact on the success of an intervention [22,23]. Continuous feedback received during a task performance is generally seen as disadvantageous for learning [22,23]. Conversely, feedback that shifts the focus outside of the body, for example, to a consequence or external visual representation of the movement, as used in this study, presumably enhances learning, even when provided very frequently [22]. However, it is not yet clear how interventions with feedback should best be designed for people with LBP [23].

In people with LBP, deficits in proprioception are suspected to contribute to consolidating changes in motor control in the long run [5] and to the reduction in postural balance [12,13]. Feedback can help overcome limitations in motor learning caused by these deficits in people with LBP [23]. Nevertheless, in a recent study investigating different feedback conditions, no differences between people with chronic LBP and people without LBP were found in learning to keep the spine in a constant position during motor control tasks [62]. In this study, practice with graphical displays of data from wearable sensors resulted in superior performance compared with practice without any feedback or practice in front of a mirror [62].

### Conclusions

We expect to gain insights into the effect of EPF from wearable sensors on postural balance, motor control, and patient-reported outcomes in people with LBP. In addition, we will estimate the extent to which people with LBP adhere to such exercising interventions when they are free to choose exercise time and frequency.

## Appendix

Multimedia Appendix 1 SPIRIT (Standard Protocol Items: Recommendations for Interventional Trials) checklist.

## Abbreviations

COP: center of pressure
EPF: exercising with postural feedback
ET: exercise therapy
ETH Zurich: Eidgenössische Technische Hochschule Zurich
LBP: low back pain
NRS: numeric rating scale
REDCap: Research Electronic Data Capture
SPIRIT: Standard Protocol Items: Recommendations for Interventional Trials
TIDieR: Template for Intervention Description and Replication
UHZ: University Hospital Zurich
